# On Some Aspects of Memory

**Published:** 1901-02-02

**Authors:** John Aikman


### ON SOME ASPECTS OF MEMORY.

By JOHN AIKMAN, M.D.

### II.—GEOGRAPHY AND TIDAL INFLUENCE.

(*Continued.*)

By a less direct channel blushing lessens the tension of blood in the brain. Both acts affect the imprint which is the basis of memory. Tears save us a record which is too heavy for us. A blush blurs the imprint of that which "it were wiser to forget." They say that a woman blushes at the first word which may indicate love, and perhaps weeps over it, and that she is pale and tearless at that which settles her future fate. Once again she may be called upon to lessen the tension on her brain by tears when the chapter closes which she has written for weal or for woe.

Infants do not shed tears till they are some months old. They do not blush until they are three or four years old, and rarely then. Idiots do not blush (Crichton Browne).

There are some soft places on the heads of young infants. This softness permits the pressure of the atmosphere to lessen the tension of the brain. As the skull hardens, the atmospheric pressure is shut off, and first the outlet of tears (the more direct channel) and then the outlet of a blush (the less direct channel) influence the tension within the skull.

When the child takes a long breath the soft parts recede, when it screams the soft parts protrude. Therefore a sigh lessens the tension within the skull; and a sigh indicates a deadlock of thought, which can be remedied by a fresh start.

The almost extinct practice of snuff-taking was said to clear the head, as a good sneeze does. Recently Dr. St. Clair Thompson and Dr. Halliburton have shown that it is probable that some of the fluid which bathes the brain escapes into the nose and throat. When such escape is prevented by the growths which sometimes exist in the nose or throat of children, those children are difficult to teach. On the other hand, a public speaker pressing his brain to its fullest tension retains this fluid within his skull, and obtains the dryness of nostril which is a help, and the dryness of throat which is a hindrance. The hungry brain learns, the full brain teaches.

### III.—ARCHITECTURE.

WE approach a larger subject when we attempt to detail the building up of memory.

I have endeavoured to show you that useful memory is the establishment of a commonweal. Whatever amateur politicians may say to the contrary, the essential of a commonwealth is not "equality." The common good is the object, but individual disparity makes up the commonweal. There is no identity in Nature—

> "So careful of the type she seems,
> So careless of the single life."
> —(Tennyson—In Memoriam, LV.)

Every blade of grass has an individuality which marks it off from its neighbour.

The philosophy which has given us a grander view of the order of creation has accustomed us to the use of the terms "development" and "developmental force." Nature perfects the whole by the diversity of its parts.

Development is progress, and it would be an outrage upon the plan of Nature if our brain cells stood still in their development. It accords with observed facts that they do not. An infant's first need is breath. The call for it is urgent, precise, and important. Some brain cells respond and a cry follows. That cry, in a less marked form, provides for the act of breathing being repeated, a variable number of times per minute, during a long life. Its next need is food and the act of swallowing is established. Already development has begun, for swallowing is a more complicated act, though it also does not imply consciousness. All that is needed is that some simple cells in the brain should have become starlike, and be capable of reflecting an impression, as a looking-glass reflects, or bends back, the sunlight upon some dark corner. But the looking-glass property, more or less simple, must be a permanent possession. To make it so changes must take place in the surface cells in the brain, which are far smaller and more closely packed than grains of sand. And the substance of the brain is so soft that many difficulties presented themselves when we endeavoured to ascertain, and show, the forms which memory cells assumed in the process of development.

It occurred to an Italian observer, Golgi by name, that as food supplied the energy, it might be possible by staining the food to note its destination. To carry out this suggestion he injected into the vessel carrying blood to the brain a solution of nitrate of silver. This substance reached and hardened the cells which were in action. So to speak, it made them fossils. But it has also a further property which has long been used in photography.

When an animal so treated was killed and thin sections of its brain were exposed to sunlight, the hardened cells turned black. We thus obtained pictures drawn by the natural process of feeding, of the changing forms which nerve cells assume under the influence of food. It remained for us to glean lessons from these pictures.

The simplest lesson you have already learnt in the description of "no memory, quick memory, and fading memory."

Those who have worked by Golgi's method tell us that the best specimens are obtained from young animals. This fact emphasises the permanence of the memories, which are most precise and most urgent. The cells which record the imprint of the need of breath, the need of food, and the sense of touch, are developed at the moment of birth. They are simple in structure, and are capable of carrying on their work without an appeal to our consciousness. They are important to the maintenance of life.

The fuller stages of development are more complex. The cells remain starlike in shape, but the offshoots are not all alike. One offshoot is a tube, continuous with a nerve fibre. It has a fairly firm sheath, and is not easily altered in shape. It represents a root; the cell body represents the trunk. The other processes have either no sheath, or one too fine to be recognised. They may be regarded as branches, and are called "dendrons" from their resemblance to trees. They are soft and capable of much variation in shape. These branches (dendrons) have still more delicate side shoots which resemble leaves or buds and are called dendrites. These buds touch or pass one another like the leaves of trees in a forest. Their contact makes or mars thought; and their movement is dependent upon some influence within the nerve cell. The movement is not a simple waving, it is a change in form and size—an expression of the fact of poverty or plenty. There is at times a winter in this forest of our brain—a condition in which "a leafless branch thy sceptre is." (Cowper Task iv. 120.)

